# Effects of weight loss rate on myostatin and follistatin dynamics in patients with obesity

**DOI:** 10.3389/fendo.2024.1418177

**Published:** 2024-06-28

**Authors:** Satoshi Kurose, Katsuko Onishi, Takumi Miyauchi, Kazuhisa Takahashi, Yutaka Kimura

**Affiliations:** ^1^ Health Science Center, Kansai Medical University, Hirakata, Osaka, Japan; ^2^ Department of Medicine II, Kansai Medical University, Hirakata, Osaka, Japan

**Keywords:** exerkine, myostatin, follistatin, physical activity, obesity, weight loss

## Abstract

**Background:**

Exercise-induced cytokines involved in controlling body composition include myostatin (MST) and follistatin (FST), both of which are influenced by physical activity. This study investigated changes in body composition and physical activity during a weight loss program, as well as the impact on serum MST and FST levels at various weight loss rates.

**Methods:**

A total of 126 patients with obesity who completed a 6-month weight loss program were divided into three groups based on weight loss rate (%): low (< 3%), middle (3–10%), and high (≥10%). The International Physical Activity Questionnaire was used for assessing physical activity, whereas dual X-ray absorptiometry was used to determine body composition. Serum MST and FST levels were measured using the enzyme-linked immunosorbent assay.

**Results:**

The middle and high groups showed a significant decrease in percent body fat and a significant increase in percent lean body mass and physical activity. Serum MST levels increased significantly in all three groups, although FST levels reduced significantly only in the middle group. After adjusting for sex and body composition, changes in peak oxygen intake (β = -0.359) and serum FST levels (β = -0.461) were identified as independent factors for the change in MST levels in the low group. Sex (β = -0.420) and changes in MST levels (β = -0.525) were identified as independent factors for the change in serum FST levels in the low group, whereas in the high group, sitting time (β = -0.600) during the weight loss program was identified as an independent factor for change in serum FST levels.

**Conclusion:**

Serum MST levels in patients with obesity increased significantly following the weight loss program, independent of weight loss rate. In contrast, serum FST levels reduced significantly only in the 3–10% weight loss group. These findings indicate that MST and FST secretion dynamics may fluctuate in response to physical activity, while also reflecting feedback regulation of body composition and metabolism during weight reduction.

## 1 Introduction

Obesity is a risk factor for cardiometabolic diseases such as hypertension, diabetes, ischemic heart disease, and stroke (1, 2). Obesity is normally treated with diet and exercise therapy; however, weight loss often leads to a decline in skeletal muscle mass as well as a reduction in body fat mass. One explanation for this phenomenon is that lower physical load caused by weight reduction reduces the absolute quantity of skeletal muscle necessary for physical activity, increasing the risk of muscle atrophy. Moreover, weight loss due to extreme energy restriction is expected to reduce muscle protein synthesis (3). Another factor that has garnered attention is the modulation of skeletal muscle mass by myokines or exercise-induced cytokines (exerkines) released by skeletal muscles (4).

Myostatin (MST) and follistatin (FST) are widely recognized exerkines involved in the regulation of skeletal muscles (5). MST, also known as growth differentiation factor 8, is a member of the transforming growth factor beta superfamily, and is secreted primarily by skeletal muscles. It acts as a negative regulator of muscle mass and inhibits muscle growth (6). Inhibition of MST contributes to suppression of muscle atrophy and reduction in fat accumulation (7). In contrast, FST, secreted by the skeletal muscle, liver, gonads, and adipose tissue, binds to MST and promotes skeletal muscle anabolism by inhibiting the myostatin/activin-ActRIIB pathway (8, 9). FST levels have been reported to increase with exercise (10). Additionally, FST expression increases in non-alcoholic fatty liver disease and plays a protective role against hepatic steatosis (11). Increased FST levels in individuals with obesity are suggested to stimulate skeletal muscle anabolism and alleviate fatty liver disease.

In our previous studies, we reported that appendicular lean mass adjusted for body weight was a positive predictor of serum MST levels and that MST significantly increased following weight loss (12, 13). We also reported that serum FST levels were positively correlated with percent body fat and negatively correlated with percent lean body mass (LBM); MST and exercise tolerance were significant negative predictors of serum FST levels (14). These findings suggest that the secretion kinetics of MST and FST may be regulated to maintain systemic homeostasis, presumably influenced by complex factors, such as changes in body composition and physical activity (15).

Variations in MST and FST kinetics could serve as potential markers for early detection and prevention of muscle atrophy during weight loss. However, given that changes in body fat and skeletal muscle vary depending on the extent of weight loss, the effect of MST and FST kinetics, along with physical activity, in response to changes in body composition remains unclear. Although previous studies have reported tissue-derived MST and FST kinetics, studies using serum samples from patients with obesity undergoing weight loss therapy are limited.

Therefore, this study aimed to investigate changes in body composition and physical activity following a weight loss program as well as their effects on serum MST and FST levels at various weight loss rates.

## 2 Materials and methods

### 2.1 Participants

The study included 126 patients with obesity who visited the obesity outpatient clinic at the Kansai Medical University Hospital between June 2014 and September 2020, and completed a 6-month weight loss program. The exclusion criteria were history of cardiac pacemaker implantation, pregnancy, severe liver dysfunction, renal disease, secondary causes of obesity owing to endocrine disorders, and wasting disease with extreme weight loss within the past 6 months.

The study was conducted in accordance with the principles of the Declaration of Helsinki, and all procedures were approved by the Ethics Committee of the Kansai Medical University (approval no. 2019092). Written informed consent was obtained from all participants before commencing the study.

### 2.2 Study protocol

This was a retrospective, observational study (**Supplementary Figure 1**). The participants finally included in the weight loss program were divided into three groups based on their weight loss rates (%): Low group, < 3%; the middle group, between > 3% and < 10%; and high group, > 10%. Data regarding clinical characteristics and past medical histories were collected from medical records. At the initial visit, lifestyle factors such as current smoking status, regular alcohol consumption, and exercise habits were determined. An exercise habit was defined as engaging in exercise for at least 150 min per week. This study compared changes in body composition, physical function, vascular function, physical activity, energy intake, and blood samples in the three groups at the end of a 6-month weight loss program. Additionally, indices associated with changes in serum MST and FST levels were analyzed for each group.

### 2.3 Weight loss program

The weight loss program was initiated once the pre-intervention evaluation was completed following the initial visit. The program, conducted under the supervision of a physician specializing in obesity, was a comprehensive 6-month program consisting of physician supervision, exercise instruction, nutritional guidance, and psychological counseling. Supervised exercise therapy was based on weekly sessions of a 60-min regimens that included stretching, 30 min of aerobic exercise at anaerobic metabolic threshold intensity, and resistance training using body weight for two sets of 10 repetitions each of three different types (lower extremity and trunk muscle). In principle, patients were instructed to perform the program, including home-based exercises, three times per week. Home-based exercise emphasized aerobic exercise and encouraged the need for greater physical activity with the goal of increasing energy expenditure. A health fitness programmer planned and instructed these workout regimens. A professional dietitian and psychotherapist provided dietary guidance and counseling on a monthly basis. The dietitian provided monthly nutritional guidance, eating behavior instruction, and dietary advice based on dietary records. The psychotherapist provided monthly counseling based on cognitive behavioral therapy, with an emphasis on self-monitoring and efficacy.

### 2.4 Measurement

#### 2.4.1 Body composition

A few days following the initial visit, body composition was measured using dual-energy X-ray absorptiometry (DXA; DPX-NT System; GE Healthcare, Buckinghamshire, UK). The measurement parameters included body weight, body fat mass, and LBM (whole body, upper extremities, trunk, and lower extremities). The percentage of body fat mass and LBM were determined by dividing by the body weight. Computed tomography and fat scan analysis software (East Japan Technology Tokyo Laboratory, Tokyo, Japan) were used to measure the umbilical visceral fat area and subcutaneous fat area.

#### 2.4.2 Physical function and arterial stiffness

Physical function was assessed based on exercise tolerance and lower limb strength. A symptom-limited cardiopulmonary test (CPX) employing a ramp protocol was performed using a cycle ergometer (AEROBIKE 75XL; Combi Co., Ltd., Japan). This regimen included an initial 4-min rest period on the ergometer, followed by 4-min warm-up at a 20-watt load, and a full workout with the load raised by 1 watt every 3–6 s to be completed within 10 min. This CPX was carried out with all patients pedaling at 50 revolutions per min. Oxygen uptake (VO_2_), carbon dioxide output, and minute ventilation on a breath-by-breath basis were measured using an expired gas analyzer (AE-300s and 310s; Minato Medical Science Co. Ltd., Japan). The anaerobic threshold was determined using the V-slope method (16). Peak VO_2_ and heart rate were defined as peak values during incremental exercise. Lower limb strength was measured twice, based on uniform rotating leg muscle strength using Strength Ergo (Mitsubishi Electric Corporation, Tokyo, Japan). The maximum value was recorded, and the weight adjustment value (N.m/kg) was used.

Arterial stiffness was assessed by measuring brachial-ankle pulse wave velocity (baPWV). After 10 min of rest in the supine position, the baPWV was measured using a pulse pressure analyzer (BP-203RPE; Omron Colin Co. Ltd., Japan). Measurements were taken twice every 2 min, and the mean value from the right and left arms was considered to be the final baPWV.

#### 2.4.3 Physical activity

Physical activity was self-reported using the shortened version of the International Physical Activity Questionnaire (IPAQ) (17). The questionnaire consisted of seven questions that assessed physical activity in the past week. Metabolic equivalent (MET) values for physical activity were classified as light (3.3MET), moderate (4.0MET), and high (8.0MET). Daily physical activity and walking time were calculated by combining physical activity, including walking for more than 10 min, with average frequency and intensity. Sitting time was defined as the average sedentary time per day between waking up to sleeping. The cutoff for evaluating the prognosis of sitting time was 480 min (18).

#### 2.4.4 Energy intake

The dietary survey consisted of self-administered food records and corresponding photographs taken over five consecutive days each month, during the weight loss program, along with interviews about dietary details. Professional dietitians recorded the names of the dishes for typical breakfasts, lunches, dinners, and snacks as well as the ingredients, weights, seasonings, and cooking methods used in those dishes. Additionally, they checked the nutritional content of the commercially procured products. From these surveys, a dietitian counted the total number of units, with 80 kcal of energy as one unit, based on Food Exchange Lists and Standard Table of Food Composition in Japan 2020 (19, 20). Daily energy intake was calculated with the formula, total units × 80 kcal.

#### 2.4.5 Blood sampling

Fasting blood samples were collected to determine the serum levels of aspartate aminotransferase, alanine aminotransferase, gamma-glutamyl transpeptidase, high-density lipoprotein cholesterol, low-density lipoprotein cholesterol, triglycerides, glucose, glycosylated hemoglobin, C-reactive protein, and serum insulin. The homeostasis model assessment of insulin resistance was calculated based on the fasting blood insulin levels at early morning, following fasting, (homeostasis model assessment of insulin resistance = [fasting serum insulin × fasting plasma glucose]/405). Blood samples were stored at −80°C. Serum exerkine (FST and MST) levels were measured using a human Quantikine ELISA Kit (R&D Systems, Minneapolis, MN, USA) according to the manufacturer’s instructions. The intra- and inter-assay coefficients of variation were 2.0–2.7% and 7.9–9.2% for FST and 1.8–5.4% and 3.6–6.0% for MST, respectively.

### 2.5 Statistical analyses

Continuous data are expressed as mean ± standard deviation or median (interquartile range), depending on the presence or absence of a normal distribution; categorical data are expressed as incidences and percentages. The Shapiro–Wilk test was used to determine the normality of the data. Comparisons between the three groups were performed using the one-way analysis of variance, Kruskal–Wallis test, or chi-squared test. The Bonferroni correction was performed as a *post-hoc* analysis. Comparisons of before and after the weight loss program were analyzed using the paired *t*-test or Wilcoxon signed-rank test, and comparisons between two groups were performed using the unpaired *t*-test. Pearson’s correlation coefficient was used to determine the relationship between changes in MST and FST, followed by multiple regression analysis. The independent variables were selected by considering the correlation and adjustment factors. All statistical analyses were performed using SPSS version 26.0 J for Windows (IBM Corp., Armonk, NY, USA), and *P*-values < 0.05 were considered statistically significant.

## 3 Results

### 3.1 Patient characteristics

The characteristics of the patients at the initial visit in the three groups are shown in **Table 1**. There were no significant differences in age, sex, body composition, or medical history between the three groups. The use of oral hypoglycemic agents in the low group was significantly higher than that in the other groups; however, there were no significant differences in terms of other medications and lifestyle habits.

**Table 1:** Patients characteristics at initial visit.

	All n = 126	Low group n = 32	Middle group n = 67	High group n = 27	P-value
Age (years)	45.9 ± 13.9	44.8 ± 13.4	47.3 ± 15.4	43.8 ± 10.2	0.483
Men, n (%)	41 (32.5)	11 (34.4)	18 (26.9)	12 (44.4)	0.250
Height (cm)	161.8 ± 8.4	161.8 ± 8.2	161.1 ± 8.3	163.7 ± 8.8	0.412
Body weight (kg)	99.2 ± 23.2	98.9 ± 22.5	97.3 ± 23.7	104.0 ± 22.8	0.457
Body mass index (kg/m^2^)	37.7 ± 7.6	37.5 ± 6.4	37.4 ± 8.0	38.8 ± 7.7	0.708
Medical history					
Hypertension, n (%)	59 (46.8)	15 (46.9)	32 (47.8)	12 (44.4)	0.958
Dyslipidemia, n (%)	35 (27.8)	9 (28.1)	19 (28.4)	7 (25.9)	0.971
Diabetes mellitus, n (%)	29 (23)	12 (37.5)	11 (16.4)	6 (22.2)	0.066
Medication					
A ntihypertensives, n (%)	46 (36.8)	13 (40.6)	23 (34.8)	10 (37.0)	0.856
Hypolipidemic agent, n (%)	31 (25.0)	9 (28.1)	16 (26.4)	6 (22.2)	0.868
Insulin, n (%)	2 (1.6)	2 (6.3)	0 (0.0)	0 (0.0)	0.054
Oral hypoglycemic agent, n (%)	26 (20.8)	12 (37.5)*	8 (12.1)	6 (22.2)	0.014
Lifestyle					
Current smoker, n (%)	9 (7.6)	5 (16.7)	2 (3.2)	2 (7.4)	0.073
Alcoholic drinks, n (%)	38 (31.9)	10 (33.3)	21 (32.8)	7 (28.0)	0.892
Exercise habits, n (%)	42 (36.8)	12 (37.5)	22 (33.8)	8 (29.6)	0.817

Data are expressed as mean ± standard deviation or median (interquartile range).

*P < 0.05 vs. Middle group.

### 3.2 Changes in physiological parameters due to the weight loss program

The changes due to the weight loss program are shown in **Table 2**, **Supplementary Table 1**. There were no significant differences in the baseline values between the three groups. Body weight, body fat mass, visceral fat area, and subcutaneous fat area significantly decreased in the middle and high groups after 6 months. The percent LBM significantly increased in the middle and high groups but significantly decreased in the low group. Furthermore, the blood pressure significantly decreased in the middle and high groups, and the baPWV significantly improved. Peak VO_2_, lower limb strength, daily physical activity, and walking time significantly increased in the middle and high groups, whereas there was significant decrease in energy intake in all groups. Aspartate aminotransferase, alanine aminotransferase, and C-reactive protein levels significantly decreased in all groups, and insulin resistance improved in the middle and high groups but worsened in the low group.

**Table 2:** Changes due to weight loss program in each group.

	Low group n = 32			Middle group n = 67			High group n = 27		
	Baseline	6-months	P-value	Baseline	6-months	P-value	Baseline	6-months	P-value
Body weight (kg)	98.3 ± 21.9	97.6 ± 22.5	0.096	96.6 ± 23.6	90.2 ± 22.5**	< 0.001	102.6 ± 22.5	89.5 ± 19.5**	< 0.001
Body fat mass (kg)	44.0 ± 13.0	44.0 ± 14.0	0.996	44.7 ± 14.7	39.6 ± 14.3**	< 0.001	45.3 ± 11.9	35.3 ± 12.9**	< 0.001
Percent body fat mass(%)	44.4 ± 6.2	44.5 ± 6.7	0.730	45.9 ± 6.3	43.4 ± 7.0**	< 0.001	44.0 ± 6.2	38.8 ± 9.3**	< 0.001
LBM (kg)	53.4 ± 11.1	52.6 ± 10.9*	0.015	51.5 ± 11.7	50.7 ± 12.7	0.063	57.1 ± 13.2	54.5 ± 11.5	0.054
Percent LBM (%)	54.9 ± 6.6	54.6 ± 6.7	0.326	53.8 ± 6.2	56.6 ± 7.1**	< 0.001	55.8 ± 6.5	61.9 ± 12.4**	0.001
Visceral fat area (cm^2^)	187.5 ± 60.1	178.7 ± 60.1	0.172	176.1 ± 64.4	153.3 ± 55.3**	< 0.001	193.6 ± 98.0	145.8 ± 89.0**	< 0.001
Subcutaneous fat area (cm^2^)	461.4 ± 195.4	444.9 ± 193.3	0.329	427.9 ± 165.3	393.4 ± 154.6**	0.001	455.1 ± 183.5	369.6 ± 155.1**	< 0.001
Systolic blood pressure (mmHg)	135.9 ± 14.3	133.1 ± 16.1	0.215	140.7 ± 18.0	134.0 ± 17.3**	< 0.001	137.9 ± 18.2	123.1 ± 13.5	< 0.001
Diastolic blood pressure (mmHg)	78.7 ± 8.3	77.0 ± 7.5	0.232	83.8 ± 12.1	77.8 ± 10.9**	< 0.001	82.3 ± 12.8	75.3 ± 9.1**	< 0.001
Rest HR (bpm)	71.8 ± 14.3	69.5 ± 12.2	0.107	72.8 ± 12.2	67.7 ± 10.1**	< 0.001	75.0 ± 10.8	62.7 ± 9.5**	< 0.001
baPWV (cm/sec)	1344.6 ± 220.7	1306.0 ± 226.2	0.097	1366.0 ± 212.4	1329.2 ± 230.2*	0.018	1311.0 ± 189.4	1250.0 ± 174.5*	0.001
Energy intake (kcal/day)	2000 (1713, 2346)	1640 (1479, 1900)**	< 0.001	1925 (1650, 2450)	1670 (1350, 2050)**	< 0.001	1795 (1500, 2344)	1513 (1288, 1975)*	0.014
Physical function									
AT (ml/kg/min)	11.0 ± 1.9	10.8 ± 1.7	0.524	11.3 ± 1.8	11.7 ± 2.1	0.063	12.2 ± 2.9	13.0 ± 4.1	0.086
Peak VO_2_ (ml/kg/min)	17.9 ± 4.1	18.0 ± 3.7	0.903	18.2 ± 3.7	19.7 ± 4.2**	< 0.001	20.2 ± 5.3	22.2 ± 6.8**	0.002
Peak HR (bpm)	142.8 ± 22.1	146.3 ± 21.9	0.210	148.3 ± 18.0	147.5 ± 21.5	0.614	148.2 ± 17.4	147.8 ± 17.8	0.859
Peak RER	1.06 ± 0.08	1.10 ± 0.09*	0.032	1.06 ± 0.09	1.09 ± 0.09*	0.029	1.06 ± 0.09	1.05 ± 0.10	0.631
Lower limb strength (Nm/kg)	1.44 ± 0.26	1.46 ± 0.30	0.306	1.48 ± 0.36	1.55 ± 0.39**	< 0.001	1.46 ± 0.34	1.64 ± 0.40**	< 0.001
Physical activity									
Daily physical activity (kcal/day)	104.0 (20.0, 523.9)	173.5 (67.7, 301.1)	0.523	123.4 (31.0, 287.9)	176.5 (72.2, 398.1)**	0.001	100.5 (0.0, 276.1)	246.3 (99.6, 650.9)**	0.022
Walking time (min/day)	25.7 (5.7, 85.7)	28.6 (14.0, 66.4)	0.268	25.7 (5.7, 40.0)	32.1 (14.0, 69.7)**	< 0.001	20.7 (0.0, 49.3)	54.3 (18.7, 100.7)**	0.005
Sitting time (min/day)	300 (180, 600)	390 (180, 585)	0.936	450 (300, 705)	420 (240, 600)	0.439	510 (300, 840)	510 (210, 720)	0.082
Biochemical examination									
AST (mg/dL)	23 (18, 37)	20 (16, 30)**	0.004	26 (19, 38)	20 (16, 26)**	< 0.001	25 (19, 35)	19 (17, 22)**	0.001
ALT (mg/dL)	30 (20, 56)	24 (17, 42)**	0.012	31 (23, 54)	23 (15, 30)**	< 0.001	22 (17, 60)	15 (13, 23)**	< 0.001
γ-GPT (mg/dL)	34 (20, 53)	30 (20, 52)	0.265	34 (25, 52)	26 (17, 36)**	< 0.001	34 (17, 54)	20 (12, 32)**	< 0.001
HDL-cholesterol (mg/dL)	43.3 ± 8.2	50.4 ± 10.1**	< 0.001	44.7 ± 11.6	53.2 ± 14.0**	< 0.001	45.1 ± 9.4	49.9 ± 13.0*	0.012
LDL-cholesterol (mg/dL)	107.9 ± 17.7	115.8 ± 17.0**	0.006	115.9 ± 30.1	119.3 ± 28.8	0.130	122.3 ± 36.8	119.9 ± 34.6	0.529
Triglycerides (mg/dL)	107 (84, 143)	102 (91, 125)	0.214	112 (79, 164)	94 (70, 135)**	< 0.001	97 (74, 129)	78 (62, 123)*	0.029
Fasting glucose (mg/dL)	110.9 ± 32.2	110.3 ± 31.3	0.922	100.2 ± 17.0	97.4 ± 10.8	0.101	98.8 ± 20.3	88.3 ± 11.8	0.008
HbA1c (%)	5.9 (5.6, 6.6)	5.9 (5.5, 6.3)	0.230	5.7 (5.5, 6.1)	5.6 (5.4, 5.9)**	< 0.001	5.6 (5.6, 6.1)	5.5 (5.3, 5.7)**	< 0.001
Insulin (µU/mL)	13.9 (8.6, 19.6)	15.5 (8.4, 22.3)*	0.013	16.1 (11.0, 19.7)	12.5 (8.8, 19.7)	0.092	12.7 (6.7, 16.6)	8.7 (6.7, 13.7)**	0.005
HOMA-IR	3.6 (2.4, 4.9)	3.9 (1.8, 6.3)	0.059	3.6 (2.5, 5.0)	3.0 (2.1, 5.2)*	0.038	2.8 (1.5, 4.8)	1.9 (1.2, 2.8)**	0.006
CRP (mg/dL)	0.23 (0.10, 0.64)	0.15 (0.07, 0.40)*	0.019	0.21 (0.08, 0.50)	0.15 (0.05, 0.46)*	0.019	0.37 (0.13, 0.57)	0.26 (0.07, 0.40)*	0.025

Data are expressed as mean ± standard deviation or median (interquartile range).

**P < 0.01, *P < 0.05 vs. significant change from baseline in each group.

LBM, lean body mass; HR, heart rate; baPWV, brachial-ankle pulse wave velocity; AT, anaerobic threshold; VO_2_, oxygen consumption; RER, respiratory exchange ratio; AST, aspartate aminotransferase; ALT, alanine aminotransferase; γ-GPT, gamma-glutamyl transpeptidase; HDL, high-density lipoprotein; LDL, low-density lipoprotein; HbA1c, glycated hemoglobin; HOMA-IR, homeostasis model assessment of insulin resistance; CRP, C-reactive protein.

**Figure 1:**
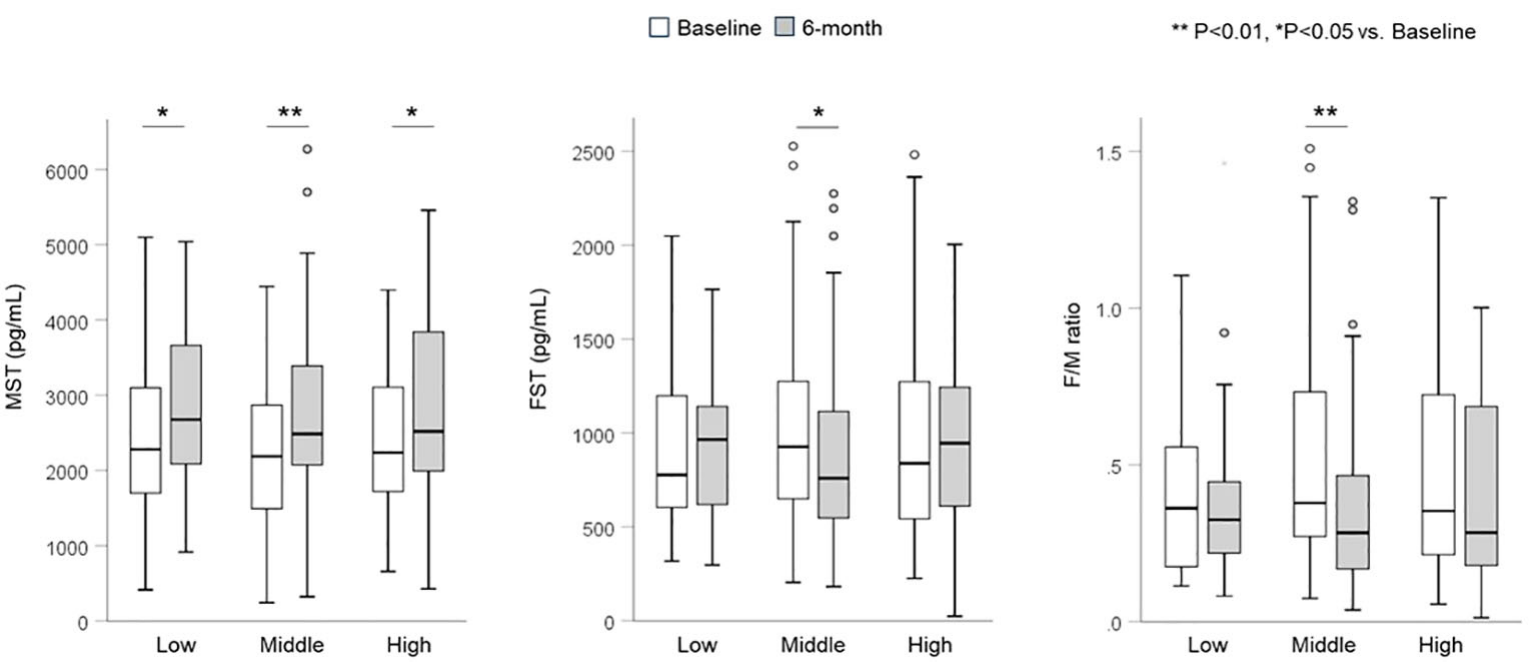
Changes in serum myostatin and follistatin levels and F/M ratio. Serum MST levels significantly increased in all three groups. Serum FST levels and the F/M ratio significantly decreased only in the middle group. FST, follistatin; MST, myostatin.

The changes in serum MST and FST levels are shown in **Figure 1**. Serum MST levels significantly increased in all groups (median, low: 2236.4 to 2519.8, middle: 2187.3 to 2484.8, high: 2279.1 to 2675.7 pg/mL) after 6 months (*P* < 0.05). However, serum FST levels (median, low: 838.4 to 945.3, middle: 926.1 to 759.0, high: 776.7 to 965.2 pg/mL) and F/M ratio (median, low: 0.35 to 0.28, middle: 0.38 to 0.28, high: 0.36 to 0.32) significantly decreased only in the middle group (*P* < 0.05). There were no significant differences in the rate of change in MST, FST, or F/M ratio between the groups.

### 3.3 Relationship between serum MST and FST dynamics and changes in body composition and physical activity

**Figure 2:**
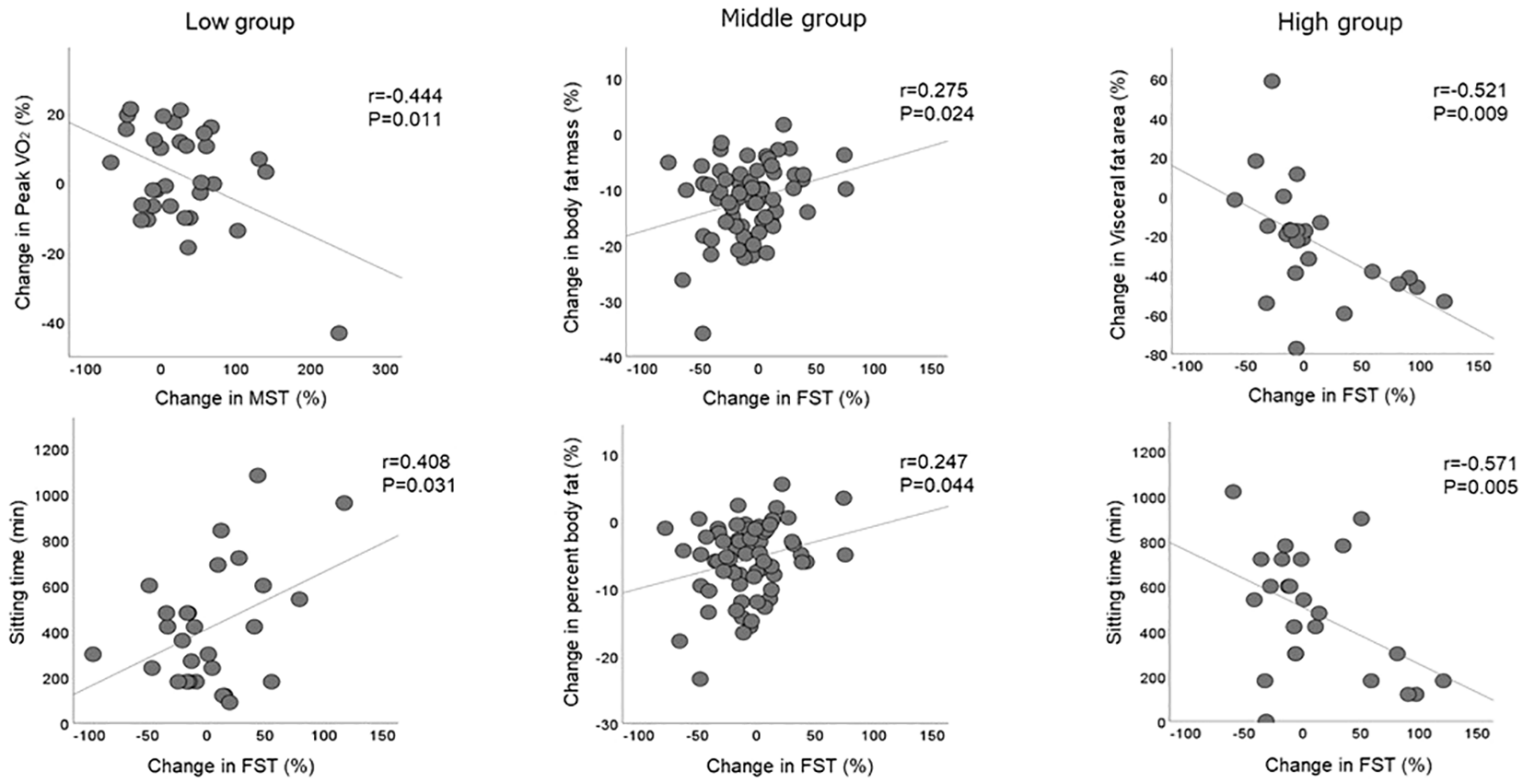
Correlation with changes in serum myostatin and follistatin levels. Significant correlations between changes in serum MST and FST levels and changes in body composition and physical activity observed within each group. FST, follistatin; MST, myostatin.

Indicators that demonstrated a significant correlation with the rate of change in serum MST and FST levels are shown in **Figure 2**. During the weight loss program, the rate of change in MST level in the low group was negatively correlated with the rate of change in peak VO_2_ (r = -0.444), whereas the rate of change in FST level was positively correlated with sitting time (r = 0.408). The rate of change in FST level in the middle group was positively correlated with the rate of body fat mass (r = 0.275) and percent body fat (r = 0.247). Conversely, the rate of change in FST levels in the high group was negatively correlated with the rate of change in the visceral fat area (r = -0.521) and sitting time (r = -0.571) during the weight loss program.

The results of the multiple regression analysis to determine the changes in serum MST and FST levels for the three groups are shown in **Table 3**. The changes in peak VO_2_ (β = -0.359) and FST levels (β = -0.461) were identified as independent factors affecting the change in MST level in the low group, even after adjustment for sex and body composition. No significant independent factors for changes in MST levels in the middle and high groups were detected. Sex (β = -0.420) and changes in MST level (β = -0.525) were identified as independent factors affecting the change in FST level in the low group, whereas sitting time (β = -0.600) during the weight loss program was an independent factor affecting the change in FST levels in the high group. No significant independent factors for changes in FST levels in the middle group were identified.

**Table 3:** Multivariate analysis to identify factors predicting changes in serum MST and FST levels.

A. MST									
	Low group			Middle group			High group		
	β	P-value	VIF	β	P-value	VIF	β	P-value	VIF
Sex	-0.307	0.072	1.212	0.037	0.810	1.313	0.099	0.737	1.347
Percent body fat (%)	0.460	0.071	2.684	0.204	0.153	1.132	0.227	0.520	1.897
Percent LBM (%)	0.169	0.453	2.253	-0.030	0.834	1.176	0.157	0.632	1.660
Peak VO_2_ (%)	-0.359	0.044	1.303	0.113	0.401	1.010	-0.111	0.690	1.193
Sitting time (min/day)	-0.055	0.781	1.751	0.192	0.172	1.103	-0.005	0.988	1.907
FST (%)	-0.461	0.017	1.462	-0.005	0.973	1.098	-0.166	0.636	1.904
B. FST									
	Low group			Middle group			High group		
	β	P-value	VIF	β	P-value	VIF	β	P-value	VIF
Sex	-0.420	0.018	1.079	0.044	0.776	1.312	0.282	0.202	1.204
Percent body fat (%)	0.155	0.581	3.102	0.154	0.290	1.152	-0.438	0.092	1.586
Percent LBM (%)	-0.088	0.715	2.301	-0.139	0.337	1.155	-0.120	0.637	1.661
Peak VO_2_ (%)	-0.266	0.175	1.450	-0.039	0.776	1.023	-0.168	0.427	1.152
Sitting time (min/day)	0.260	0.208	1.627	0.142	0.321	1.122	-0.600	0.013	1.209
MST (%)	-0.525	0.017	1.664	-0.005	0.973	1.119	-0.099	0.636	1.129

β standardize partial regression coefficient.

Sex: men = 1, women = 2.

VIF, variance inflation factor; LBM, lean body mass; VO_2_, oxygen consumption; FST, follistatin; MST, myostatin.

Percent body fat, percent lean body mass, peak VO_2_, FST and MST were input as percent changes before and after the program.

**Figure 3:**
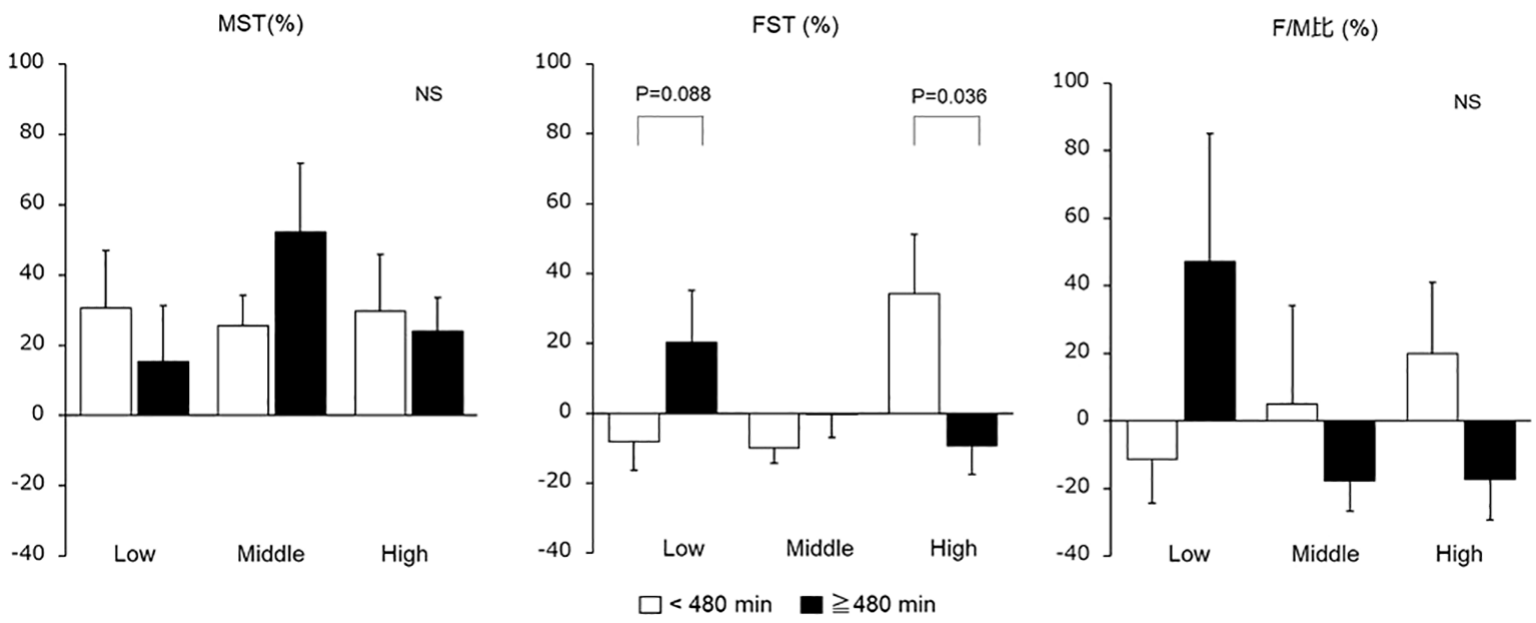
Changes in serum myostatin and follistatin levels and F/M ratio due to sitting time. Changes in serum MST and FST values and F/M ratio were compared by classifying the daily sitting time of each group as 480 min. FST, follistatin; MST, myostatin; NS, not significant.

The MST, FST, and F/M ratio were compared by classifying the sitting time of each group with a 480 min cutoff, revealing that the serum FST levels in the high group were significantly increased with < 480 min of sitting (**Figure 3**).

## 4 Discussion

This was a retrospective study conducted in patients with obesity who completed a 6-month weight loss program, and examined the dynamics of serum MST and FST levels under conditions confirming changes in body composition and metabolism, similar to previous reports on weight loss effects (21). The results showed that serum MST levels significantly increased, regardless of the extent of weight loss, whereas serum FST levels significantly decreased only when the weight loss was 3–10%. Together with variations in the exerkine levels, LBM in the low group significantly decreased, whereas the middle and high groups showed no significant change. Additionally, the middle and high groups experienced a significant increase in LBM percentage. The exercise regimen in this study was not sufficient to increase the total amount of LBM during weight loss, however the increase in LBM percentage in the middle and low groups may be attributed to the reduction in fat mass associated with weight loss. Although serum MST levels significantly increased in all the groups after the weight loss program, these changes may have different underlying mechanisms. The low group showed almost no changes in body composition or physical activity, whereas their insulin resistance tended to worsen. Individuals with obesity have been reported to show a positive association between high MST and high serum insulin levels, regardless of skeletal muscle mass, suggesting that the effects of worsening insulin resistance may be a part of the mechanism underlying the increase in MST levels in the low group (22). We also found that the change in peak VO_2_ was a negative independent factor for the change in MST levels in the low group. In a previous study, regular aerobic training, whereas adjusting dietary intake to prevent changes in body composition, significantly decreased MST levels and increased exercise tolerance (23). Regular exercise has been reported to contribute to a decrease in MST levels, whereas physical inactivity may contribute to skeletal muscle atrophy by inducing an increase in MST levels (24, 25). Therefore, the increase in MST level in the low group was thought to be influenced by worsening insulin resistance and physical inactivity. The middle and high groups showed significant decreases in percent body fat and insulin resistance, coupled with notable increases in percent LBM, physical activity, and exercise tolerance. Although increased physical activity contributes to a decrease in MST levels (24–27), MST is also expected to inhibit skeletal muscle synthesis to maintain homeostasis in response to successful weight loss and increased percent LBM. Our previous study also reported a positive association between high MST levels and high appendicular lean mass in patients with obesity (12). This suggests that the increase in MST levels observed in the middle and high groups may be attributable to an inhibitory response of muscle synthesis heightened skeletal muscle mass relative to body weight. Changes in body composition, particularly percent LBM, may have greater impact on MST dynamics in individuals with obesity than physical exercise.

In contrast, serum FST levels decreased significantly only in the middle group after the weight loss program. The FST level is known to increase with exercise, which contributes to its beneficial effects. FST is secreted by the liver and has been recognized for its potential therapeutic effects in fatty liver disease. It can help improve liver function and reduce liver fat accumulation, enhancing compensation in fatty liver conditions (11, 23, 28). We reported higher serum FST levels in patients with severe obesity than in those with obesity defined as a body mass index <35 kg/m^2^ (14). Other studies have also reported elevated FST levels in individuals with obesity compared with healthy individuals (29). Since the low group in this study did not demonstrate any changes in body composition or physical activity, it is expected that fatty liver and hepatic insulin resistance due to obesity did not improve. Thus, serum FST levels in the low group was unlikely to exhibit any significant difference after the weight loss program. Serum FST levels in the middle group exhibited a positive correlation with body fat and improved liver function while also showing a significant increase in physical activity and exercise tolerance. The details of the mechanism are unclear; however, it is possible that the compensatory increase in FST levels was reduced by improvements in fatty liver and increased physical activity associated with weight loss. Our results for the middle group are consistent with those of previous studies that demonstrated decrease in FST levels with weight loss and reduction in FST being associated with changes in body fat (30). However, the serum FST levels in the high group were not significantly different after the weight loss program. The high group experienced a significant weight loss (>10%), and the decreasing FST, like the trend shown in the middle group, may have reappeared due to other causes. The high group exhibited a more strongly optimized body composition and liver function than the low and middle groups and had increased physical activity and exercise tolerance. In addition, the multivariate analysis showed that an increase in FST levels in the high group was associated with sedentary time during the weight loss program. Long periods of sitting are likely to have an impact on exerkine kinetics since muscle activity during sedentary behavior is lower than during standing and walking (31). Though the IPAQ used in this study may have low reliability in assessing walking and overall physical activity levels, and may not be able to assess detailed activities within 10 min, the assessment of sitting time has been reported to be moderately reliable (32). When weight reduction is successful and the weight loss rate is high, even low-intensity and brief muscle stimulation that is not reflected in the physical activity questionnaire may have a positive effect on serum FST levels. These findings suggest that the compensatory increase in FST levels in patients with obesity may decrease with weight loss and that further improvement in body composition and physical activity may lead to the desired increase in FST levels.

The strength of this study was its ability to confirm the variability in serum MST and FST levels at various weight loss rates. Earlier studies reported either no change, or a decrease in MST levels and an increase in FST levels with exercise therapy (24–27, 33). In contrast, our findings indicate an overall increase in MST levels before and after the weight loss program. Since this weight loss program was a combination of exercise and diet therapy, it is difficult to attribute these findings solely to the effects of exercise or physical activity on exerkines. It is more likely that the effects of exercise and physical activity are modified by concurrent changes in body composition. An important finding of this study is that physical activity, particularly changes in exercise tolerance, has a direct effect on MST and FST dynamics under conditions where there are no changes in body composition. However, it is challenging to explain the variations in MST and FST solely based on physical activity because of feedback regulation resulting from changes in body fat and skeletal muscle when the weight loss is ≥ 3%. MST and FST are exerkines that regulate skeletal muscle mass, and it has been suggested that they orchestrate changes in body composition, metabolism, and physical activity. Although there are numerous challenges in understanding skeletal muscle anabolism and catabolism based on the dynamics of MST and FST during weight loss, these exerkines may serve as potential early indicators for promoting optimal weight loss.

This study had several limitations. First, patients were retrospectively classified into three groups according to weight loss rate, and changes in serum MST and FST levels due to stepwise weight loss within the same patients could not be evaluated. Previous studies have reported the effects of rate of weight loss on skeletal muscle mass and MST (30). Therefore, longitudinal observations of the changes in MST and FST secretions associated with the changes in body weight are required in future research. Second, this study evaluated MST and FST using serum samples, and the detailed mechanisms remain unknown because the serum is a mixture of secretions from various organs in addition to skeletal muscle. However, longitudinal studies of human muscle tissue are ethically challenging. Hence, it is important to elucidate variations in myokines involved in skeletal muscle regulation in response to changes induced by serum. Third, we could not exclude sex-related differences. Previous studies have reported sex differences in MST and FST (34), indicating the importance of increasing the sample size and conducting separate analyses for men and women in future studies. Fourth, the study did not include a control group owing to ethical concerns regarding not implementing a weight loss program for these patients with obesity. Although there was no statically significant difference in baseline value between the three groups, a range of values was observed. This could be due to the complex influence of contextual factors, including the duration of obesity, treatment experience, education, and personality. Finally, detailed assessments of dietary intake such as protein intake were not performed. Nevertheless, no significant differences in energy intake were observed among the three groups, suggesting that the extent of weight loss was predominantly influenced by physical activity and exercise.

In conclusion, serum MST levels in patients with obesity significantly increased before and after the weight loss program, regardless of the rate of weight loss. However, serum FST levels significantly decreased only when the weight loss rate was between 3–10%. These changes in myokines may have different implications for the different rates of weight loss. Specifically, in situations in which the body composition of individuals with obesity remains unchanged, this may reflect the influence of physical activity and exercise. However, when changes in body composition are accompanied by weight loss, feedback regulation resulting from alterations in body fat mass, skeletal muscle mass, and metabolism may also play a role in addition to physical activity, suggesting a potential impact on these secretion dynamics.

## Data availability statement

The raw data supporting the conclusions of this article will be made available by the authors, without undue reservation.

## Ethics statement

The studies involving humans were approved by the Ethics committee of the Kansai Medical University. The studies were conducted in accordance with the local legislation and institutional requirements. The participants provided their written informed consent to participate in this study.

## Author contributions

SK: Writing – original draft, Writing – review & editing, Conceptualization, Data curation, Formal Analysis, Investigation, Methodology. KO: Data curation, Formal Analysis, Validation, Writing – review & editing. TM: Investigation, Project administration, Writing – review & editing. KT: Investigation, Supervision, Writing – review & editing. YK: Conceptualization, Investigation, Methodology, Project administration, Supervision, Writing – review & editing.
