# Supplementary material for: Effects of weight loss rate on myostatin and follistatin dynamics in patients with obesity
Source: Front Endocrinol (Lausanne). 2024 Jun 28;15:1418177. doi: 10.3389/fendo.2024.1418177 (PMC11239380; doi:10.3389/fendo.2024.1418177)
Supplement: Supplementary file 1 [file DataSheet_1.docx]

Supplementary Material

# Supplementary Figures and Tables

## Supplementary Figures

**
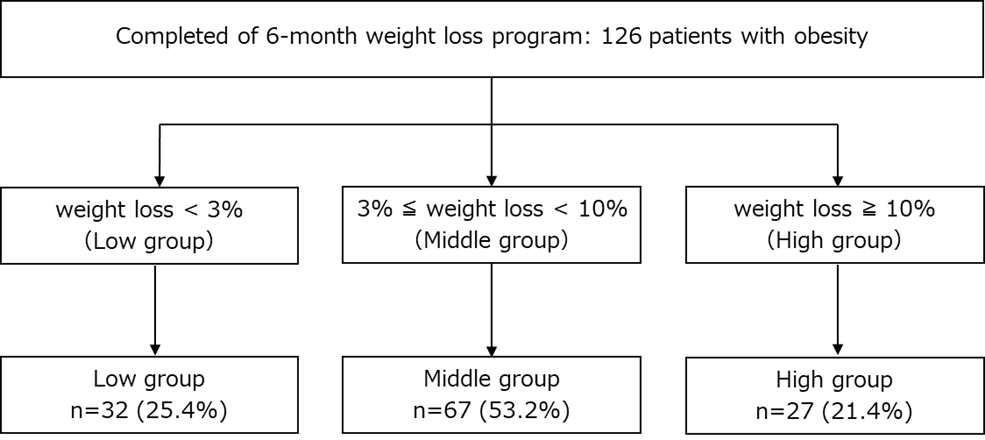
**

**Supplementary Figure 1.** **Study protocol**

A total of 126 patients with obesity who completed a 6-month weight loss program were divided into three groups.

## Supplementary Tables

**Supplemental Table 1. Comparison of changes in rates owing to 6-month weight loss program in each group**

Data are expressed as median (interquartile range).

** P < 0.01, * P < 0.05 vs. Low group, ‡ P < 0.01, † P < 0.05 vs. Middle group

HR, heart rate; baPWV, brachial-ankle pulse wave velocity; AT, anaerobic threshold; VO2, oxygen consumption; RER, respiratoty exchange ratio; AST, aspartate aminotransferase; ALT, alanine aminotransferase; γ-GPT, gamma-glutamyl transpeptidase); HDL, high-density lipoprotein; LDL, low-density lipoprotein; HbA1c, hemoglobin A1c; IRI, immunoreactive insulin; HOMA-IR, homeostasis model assessment of insulin resistance; CRP, C-reactive protein.
